# The Anatomy of connection: Rethinking communication beyond the script

**DOI:** 10.1017/S1478951525101235

**Published:** 2025-12-23

**Authors:** João Carlos Geber-Junior, Daniel Neves Forte

**Affiliations:** 1 Center for Oncologic Intercurrence Care (CAIO), Cancer Institute of the State of São Paulo (ICESP), São Paulo, Brazil; 2 Faculty of Health Sciences, Sírio-Libanês Hospital, São Paulo, Brazil; 3 Hospital das Clínicas, Faculty of Medicine, University of São Paulo (FMUSP), São Paulo, Brazil

The COVID-19 pandemic disrupted more than healthcare systems – it fractured the fragile architecture of communication between physicians, patients, and families. As a resident in Internal Medicine at one of the largest COVID-19 referral centers in Latin America, I witnessed not only clinical collapse but the disintegration of trust and relational presence, especially in high-stakes conversations involving prognosis, dying, and care decisions.

Amid overflowing ICUs, grieving families, exhausted colleagues, and a nation engulfed in political discord and misinformation, one reality became inescapable: our formal protocols for delivering bad news – particularly the SPIKES model – were no longer enough (Back et al. 2020). I had been introduced to SPIKES as a medical student through countless role-plays and classroom simulations. It offered structure and represented a welcome advance over the detached biomedical model. But during the pandemic, the model faltered.

Masked, fully gowned, and often speaking through screens or tablet devices, I was expected to embody empathy and compassion while reporting a patient’s death to loved ones who had not seen their relative for days. “Setting” was no longer a quiet room but a noisy hallway or a personal protective equipment–donning station. Asking about a family’s “Perception” or inviting them into a conversation felt hurried and artificial. Delivering “Knowledge” – the core of the message – occurred without visual contact or physical presence. The “Emotions” phase, once rooted in shared affect and silence, was reduced to desperate cries on the other side of a screen. And the final step – “Strategy and Summary” – offered little relief, as there were often no plans left to make. On the front lines, SPIKES increasingly felt like a list of frustrated intentions – less a guide to compassionate dialogue than a painful reminder of what the crisis had rendered unattainable (Back et al. 2020).

In July 2020, I became a patient myself. Alone in a new city, recently admitted to residency, I developed moderate COVID-19 with rapidly worsening dyspnea and desaturation. I recall watching my own oxygen saturation drop to 80% on room air. With no family nearby, I depended on the kindness of mentors who left food and medicine at my door. I survived. But the experience of solitary vulnerability – of physical decline without witness – etched in me a deeper awareness of what it means to be truly seen, heard, and accompanied.

It was only five years later, in 2025, during my specialization in Palliative Care, that I encountered a new model of communication – one that helped me make sense of what had failed. Introduced in a class titled “The Anatomy of Communication,” the approach proposed a hierarchy grounded in neurobiology and clinical practice: trust, emotion, and shared cognition (Forte et al. 2024). More than a protocol, it was a relational map for navigating difficult conversations – not linearly, but fluidly, in resonance with the patient’s emotional state and the clinician’s own regulation.

Trust, as the first layer, is not built through verbal explanation. It emerges from presence – conveyed through body language, eye contact, posture, and congruence. Establishing trust is not a preliminary step; it is the foundation of every word that follows. Yet during the pandemic, the very conditions required to convey trust were stripped away. Without proximity, without facial expressions, with time constrained and systems overwhelmed, visual contact—often through video calls rather than audio alone—became a grounding starting point, allowing for an honest conversation and the establishment of a shared commitment: “I will tell you the truth, and we are here to work for what is best for you”.

The second layer – emotion – requires not merely acknowledgment but engagement with the affective dimension of suffering. This calls on clinicians to tolerate ambiguity, absorb emotional dissonance, and regulate their own responses while prioritizing the patient’s emotional agenda. Empathy, in this frame, is not sentimental. It is attunement, anchored in the neurobiological phenomenon of emotional resonance. This explains why moments of silence or simple gestures may carry more meaning than elaborate explanations. But it also demands that we remain whole while witnessing another’s fracture.

Only once trust and emotion have been addressed can we enter the third layer: shared cognition. Prognostic disclosure, advance care planning, and therapeutic decisions belong here – not as a starting point, but as a negotiated space built upon relational grounding. This model acknowledges that conversations oscillate. They circle back, pause, restart, and evolve. This is not a flaw – it is the nature of human dialogue under strain. The approach echoes the insights of Jackson and Emanuel, who emphasize that communication with seriously ill patients must be iterative, emotionally attuned, and rooted in trust to support meaning-making and shared decision-making (Jackson and Emanuel 2024).

What moved me most about this model was its biological and anthropological grounding. Empathic behaviors are not exclusive to humans; they are observed in primates, dogs, even rodents. Our capacity to console, to bear witness to suffering, is evolutionarily wired. Cultural context matters as well. In Brazil, where relationality and spirituality permeate everyday life, communication is often infused with metaphors of hope. When a family speaks of miracles, they are not necessarily in denial – they are expressing resilience. Countering that hope with data is not only ineffective; it may constitute a symbolic rupture in the therapeutic relationship. Embracing that hope, even without sharing it, can be the bridge to authentic dialogue. This balance between honesty and hope has long been emphasized in the palliative care literature (Forte et al. 2024).

In contexts where patients and families are navigating existential terrain, our communication must be more than transactional – it must be transformative. What we need is not a replacement for SPIKES, but a reconceptualization of its goals. Communication in serious illness is not merely the transfer of information – it is the co-creation of meaning. And meaning is born not from content, but from connection.

The pandemic exposed the fragility of our clinical scripts and the magnitude of our emotional debt. But it also opened a space for innovation – anchored not in technology, but in presence. The next revolution in medicine may not lie in algorithmic precision or digital access, but in our capacity for relational depth.
